# Longitudinal symptomatic interactions in long-standing schizophrenia: a novel five-point analysis based on directed acyclic graphs

**DOI:** 10.1017/S0033291721002920

**Published:** 2023-03

**Authors:** Giusi Moffa, Jack Kuipers, Giuseppe Carrà, Cristina Crocamo, Elizabeth Kuipers, Matthias Angermeyer, Traolach Brugha, Mondher Toumi, Paul Bebbington

**Affiliations:** 1 Department of Mathematics and Computer Science, University of Basel, Basel, Switzerland; 2 Division of Psychiatry, University College London, 149 Tottenham Court Road, London W1T 7NF, UK; 3 D-BSSE, ETH Zurich, Basel, Switzerland; 4 Department of Medicine and Surgery, University of Milano Bicocca, Via Cadore 48, Monza 20900, Italy; 5 Department of Psychology, IoPPN, King's College London, London SE5 8AF, UK; 6 Department of Psychiatry, University of Leipzig, Johannisallee 20, 04137 Leipzig, Germany; 7 Department of Health Sciences, College of Life Sciences, University of Leicester, Centre for Medicine, University Road, Leicester LE1 7RH, UK; 8 Laboratoire de Santé Publique, Université de la Méditerranée, Marseille, France

**Keywords:** Affect, Bayesian analysis, longitudinal studies, negative symptoms, network models, positive symptoms, schizophrenia

## Abstract

**Background:**

Recent network models propose that mutual interaction between symptoms has an important bearing on the onset of schizophrenic disorder. In particular, cross-sectional studies suggest that affective symptoms may influence the emergence of psychotic symptoms. However, longitudinal analysis offers a more compelling test for causation: the European Schizophrenia Cohort (EuroSC) provides data suitable for this purpose. We predicted that the persistence of psychotic symptoms would be driven by the continuing presence of affective disturbance.

**Methods:**

EuroSC included 1208 patients randomly sampled from outpatient services in France, Germany and the UK. Initial measures of psychotic and affective symptoms were repeated four times at 6-month intervals, thereby furnishing five time-points. To examine interactions between symptoms both within and between time-slices, we adopted a novel technique for modelling longitudinal data in psychiatry. This was a form of Bayesian network analysis that involved learning dynamic directed acyclic graphs (DAGs).

**Results:**

Our DAG analysis suggests that the main drivers of symptoms in this long-term sample were delusions and paranoid thinking. These led to affective disturbance, not vice versa as we initially predicted. The enduring relationship between symptoms was unaffected by whether patients were receiving first- or second-generation antipsychotic medication.

**Conclusions:**

In this cohort of people with chronic schizophrenia treated with medication, symptoms were essentially stable over long periods. However, affective symptoms appeared driven by the persistence of delusions and persecutory thinking, a finding not previously reported. Although our findings as ever remain hostage to unmeasured confounders, these enduring psychotic symptoms might nevertheless be appropriate candidates for directly targeted psychological interventions.

## Introduction

Early studies of severe mental disorders distinguished those in which abnormalities of perception and interpretation seemed of central importance, from those where the salient abnormalities reflected mood disturbance. This formed the basis of Kraepelin's dichotomy between manic depressive illness and dementia praecox (otherwise schizophrenia). However, cross-sectional and longitudinal associations of psychotic experiences with anxiety and depression are now well established (e.g. Ben-Zeev, Ellington, Swendsen, & Granholm, 2011; Krabbendam *et al*., 2004; Quattrone *et al*., 2019; Sax *et al*., 1996; Scott *et al*., 2009; Smith *et al*., 2006; Thewissen *et al*., 2011; Upthegrove, Marwaha, & Birchwood, 2017; Yung *et al*., 2007), whereas a smaller number of studies indicate similar findings for low self-esteem, worry and sleep disturbance (e.g. Freeman *et al*. 2012; Hartley, Haddock, Vasconcelos E Sa, Emsley, & Barrowclough, 2014; Sheaves *et al*. 2016). These affective changes were initially explained as a secondary response to the impact of schizophrenia on social status and circumstances (Birchwood, Iqbal, & Upthegrove, 2005). However, they might equally reflect mediation of the emergence of psychotic phenomena subsequent to psychosocial trauma (Hardy et al., 2016; Marwaha & Bebbington, 2015).

Whatever the mechanisms, depression and anxiety regularly precede the earliest stages of psychosis (Bird, Evans, Waite, Loe, & Freeman, 2019; Freeman, Taylor, Molodynski, & Waite, 2019; Fusar-Poli, Nelson, Valmaggia, Yung, & McGuire, 2014) and a propensity to affective dysregulation may therefore be intrinsic to the schizophrenic process (Marwaha, Broome, Bebbington, Kuipers, & Freeman, 2014). Thus, symptoms of low self-esteem, worry, sleep disturbance, anxiety and depression may constitute contributory causes of specific psychotic symptoms, especially paranoia (e.g. Bebbington *et al*., 2013; Freeman, 2016; Kuipers *et al*., 2006). Damaging social experiences also seem to be implicated in the development of psychotic phenomena (e.g. Bebbington *et al*., 2011; Hailes, Ronggin, Danese, & Fazel, 2019; Kelleher *et al*., 2013; McGrath *et al*., 2017). This relationship might in turn be mediated or moderated by non-psychotic symptoms (e.g. Gibson, Alloy, & Ellman, 2016; Gracie *et al*., 2007; Marwaha & Bebbington, 2015). This has clinical relevance, as such symptoms might then serve as significant targets for psychological or pharmacological treatments. Finally, although psychoactive substances such as cannabis may elicit paranoia by directly inducing anomalous perceptual experiences such as hallucinations, they may also operate through these common affective processes (Freeman et al., 2015).

Such interactional models are difficult to analyse. However, recent advances in statistical methods allow stronger inferences about complex causal relationships. Applied to mental disorders, these treat the pattern of symptoms as a causal system of individual interacting variables (Borsboom & Cramer, 2013; Fried et al., 2017; Isvoranu et al., 2017; Isvoranu, Borsboom, van Os, & Guloksuz, 2016; Klippel et al., 2017; McNally, 2016; McNally et al., 2015; Murphy et al., 2017; Wigman, de Vos, Wichers, van Os, & Bartels-Velthuis, 2017). Several methods are available for such analyses. They include association networks, partial correlation networks and relative importance networks (McNally, 2016). All describe the strength and the relative importance of different covariates in the prediction, but not causation. We have therefore argued for the use in psychological research of probabilistic graphical models based on directed acyclic graphs (DAGs). These provide improved insights into putative causal links, and allow us to represent the full picture in one unique model. In this way, we can more easily appreciate the complexity of the mechanisms linking the variables to each other (Kuipers, Moffa, Kuipers, Freeman, & Bebbington, 2019; Moffa et al., 2017).

DAGs are graphical structures underlying Bayesian networks modelling the overall dependence structure of multiple variables. The specific variables we wish to analyse are represented as *nodes* connected by directed *edges*, and these together form a network (see Moffa et al., 2017 for a more detailed description). Kuipers and Moffa (2017) introduced a novel Bayesian method for learning DAG structures and have more recently extended it to achieve greater efficiency (Kuipers et al., 2018; https://arxiv.org/abs/1803.07859). The Bayesian approach to learning DAG structures coupled with Pearl's *do calculus* (Pearl, 2009) allows us to derive ranges of putative causal effects. We have previously used this strategy to demonstrate the role of affective change in mechanisms linking bullying with persecutory ideation and hallucinations (Moffa et al., 2017).

Bayesian networks can also be useful for modelling the overall dependence structure of multiple variables measured over *different* time-points. In such *dynamic Bayesian networks* (*DBNs*), we may refer to the individual waves of data collection as *time-slices*: there are thus nodes for each variable in each time-slice. Nodes may be connected within a time-slice, but may also be linked to variables in the follow-up time-slice, thereby forming a larger DAG structure incorporating measurements of all variables over time. We have previously applied this form of Bayesian network analysis to initial and 18-month follow-up data from the 2000 British National Psychiatric Morbidity survey in order to evaluate the interaction between non-psychotic and psychotic phenomena over time (Kuipers et al., 2019).

Schizophrenia is classically a chronic condition, and even with modern clinical management and the consistent deployment of antipsychotic medication some sufferers certainly experience enduring and disabling symptoms. Establishing the mechanisms of symptom persistence may suggest new initiatives for treatment. An opportunity to do this is provided by the large European Schizophrenia Cohort (EuroSC) comprising people with longstanding difficulties in contact with services, and in receipt of prescribed medication (Bebbington et al., 2005). Detailed symptom information was obtained at five time-points over a 2-year period. The current analysis applies DAGs to this longitudinal dataset in a novel application of this methodology.

We hypothesised that, in line with mechanisms found to precede acute psychotic disorders, affective disturbance in the form of anxiety and depression would exert an important and consistent effect on the persistence of delusions and hallucinations.

## Methods

### Participants

EuroSC was a naturalistic 2-year follow-up of a cohort of people aged 18–64, with an established diagnosis of schizophrenia. They were recruited from secondary psychiatric services in nine community mental health catchment areas, three in France, four in Germany and two in the UK. Ethical approval for the study was obtained locally in each country. Their illness was longstanding, averaging 16 years in duration at intake. Ethical approval for the study was obtained locally in each jurisdiction. Eligible patients were aged 18–64 years at the time of enrolment, had a diagnosis of schizophrenia according to DSM-IV criteria, and had given signed informed consent. Only those remaining in the established programmes of psychiatric care were followed up, and treatment was centred on prescribed medication. The diagnosis of schizophrenia and the assessment of positive and negative symptoms were based on systematic semi-structured interviews. Recently hospitalised subjects were excluded, and the sample was consequently not acutely unwell. However, participants remained significantly symptomatic. The survey provides detailed information on clinical symptoms at five time-points. In total, 1208 people with schizophrenia entered the study, 288 in France, 302 in the UK and 618 in Germany.

However, the primary objective of EuroSC was to identify and describe the types of treatment and patterns of care seen in people with schizophrenia, and to relate these to clinical outcomes.

### Measures

An extensive battery of instruments was used to collect information during face-to-face interviews. In the UK and Germany, SCAN (Schedules for Clinical Assessment in Neuropsychiatry-version 1.0; WHO, 1992) was used to evaluate the 4-week period before interview and the most significant period of earlier psychopathology. Its component algorithm then allowed the establishment of research diagnoses of schizophrenia. In the French centres, schizophrenia was identified using the Structured Clinical Interview for DSM-IV (SCID; Spitzer, Williams, Gibbon, and First, 1992).

For the purpose of the current analyses, we selected items from the interviewer-administered Positive and Negative Syndrome Scale (PANSS) (Norman, Malla, Cortese, & Diaz, 1996): P1, delusions; P3, hallucinations; P4, excitement; P5, grandiosity; P6, suspiciousness; P7, hostility; G2, anxiety; G11, poor attention and G14, poor impulse control. These were rated in relation to the preceding 72 h, and our analysis was based on items scored as being clearly present and of moderate severity (a rating of 4 or above). We also sourced items from the Calgary Depression Scale for Schizophrenia (CDSS) (Addington, Addington, & Schissel, 1990; Addington, Addington, Maticka-Tyndale, & Joyce, 1992): item 1, depressed mood; item 2, hopelessness and item 3, self-depreciation. This choice of items was derived from our initial hypothesis that affective symptoms have a significant role in the emergence and maintenance of psychotic symptoms.

### Procedures

Trained research assistants obtained informed consent from participants, while reassuring them about privacy protection. The current analysis uses data from all five time-points. We term the interval between adjacent time-points a *time-slice*.

Inevitably some participants were lost to follow-up, progressively so during the course of the study, such that only 810 were available for assessment by the fifth time-point. However, attrition analyses showed no significant differences in demographic factors or in any of the study variables between participants with data missing at any follow-up and the participants who remained in the study throughout (Carrà et al., 2020). We were able to include in the analysis all participants with complete data across any two adjacent time-points. The data for the prior time-point then contribute to learning the internal structure of the time-slice. All participants with observations at two adjacent time-points contributed to learning the connections between time-slices, whereas those with available data for multiple pairs of adjacent time-points contributed to the analysis once for each pair.

For the sociodemographic characteristics of the sample, see Table 1. The scores on PANSS and the CDSS over the five datapoints are shown in Table 2, together with the exact number of participants included each time. Overall, the mean scores for the psychotic symptoms and depression measures showed statistically non-significant reductions with time (PANSS positive 12.4 to 11.3; PANSS negative 15.8 to 15.1; PANSS General 29.3 to 26.6; CDSS 2.9 to 2.1).

**Table 1.** Sociodemographic and clinical characteristics at baseline

	*N* = 1208
Male	743 (62)
Married/living as couple	254 (21)
Living condition	
Alone	417 (35)
With partner	268 (22)
With family	295 (24)
Collective accommodation	141 (12)
Homeless	87 (7)
Ever worked	1107 (92)
Age (years), mean (s.d.)	40.76 (10.97)
Education (years), mean (s.d.)	10.12 (2.11)
Age of illness onset (years), mean (s.d.)	26.25 (8.37)
Length of illness (years), mean (s.d.)	15.66 (9.81)
Overall illness course	
Single episode, full remission	46 (4)
Single episode, partial remission	45 (4)
Episode – no symptoms	235 (20)
Episode – residual symptoms	500 (42)
Continuous	304 (26)
Other or unspecified pattern	61 (5)
With prominent negative symptoms	395 (33)
Antipsychotic medication	
FGAs	702 (58)
SGAs	291 (24)
FGAs + SGAs	159 (13)
Antidepressant medication	159 (13)
PANSS score, mean (s.d.)	
Positive	12.39 (5.57)
Negative	15.76 (7.64)
Gen Psych	29.33 (10.68)
CDSS score, mean (s.d.)	2.91 (3.58)

s.d., standard deviation; FGA, first-generation antipsychotics; SGA: second-generation antipsychotics; PANSS, Positive And Negative Syndrome Scale; CDSS, Calgary Depression Scale for Schizophrenia.

Values in parentheses are percentages except as otherwise indicated.

**Table 2.** Mean (s.d.) for PANSS and CDSS total scores and participant numbers at all five time-points

	Time 1 (baseline) *N* = 1208	Time 2 (6 months) *N* = 1024	Time 3 (12 months) *N* = 962	Time 4 (18 months) *N* = 861	Time 5 (24 months) *N* = 810
PANSS scores					
Positive	12.39 (5.57)	11.96 (5.53)	11.84 (5.24)	11.56 (5.34)	11.31 (5.26)
Negative	15.76 (7.64)	15.53 (7.14)	15.49 (7.29)	15.28 (7.11)	15.06 (7.20)
Gen Psych	29.33 (10.68)	28.18 (9.93)	28.16 (9.53)	27.22 (9.47)	26.57 (9.15)
CDSS score	2.91 (3.58)	2.44 (3.46)	2.37 (3.33)	2.26 (3.23)	2.07 (3.25)

PANSS, Positive And Negative Syndrome Scale; CDSS, Calgary Depression Scale for Schizophrenia.

### Statistical methods

As the data provide repeated measurements at five successive time-points, we had access to the relationship between an antecedent data-point and its immediate successor 6 months later in relation to four separate intervals. Combining the measurements of all symptoms in one model allows us to capture the mechanisms linking the variables to each other in a single representation, and informs us about potential causal effects between a range of symptoms on a 6 month scale.

We accordingly used *dynamic Bayesian networks* to capture the time component and to describe the evolution of the variables (Friedman, Murphy, & Russell, 1998). Such networks model the overall dependence structure of multiple variables, measured over different time-points, and can be visualised in DAGs (see online Supplementary Fig. S1 for a toy example). In their simplest form, DBNs assume that the connections within a time-slice are stationary over time and identical across time-slices, implying that the way symptoms relate to each other does not change. Essentially, the requirement for stationarity over time and identical connections across time-slices translate into the assumption that the way in which symptoms *interact*, with each other and over time, does not change substantially with disease progression (these assumptions receive tangential support from the previous attrition analysis: Carrà et al., 2020).

The problem of inferring the network that describes the observed data is essentially reduced to learning a network of size equivalent to the number of variables considered at each time-point. For each variable in the follow-up time-slice, we also need to learn the set of parents from the antecedent time-slice [i.e. the variables from the previous time-point with a direct link (*edge*) to the variable under consideration, for which the data rejected all possible conditional independence relationships]. The computational cost of identifying the models characterising the data well does not increase significantly. Using DBNs allows us to characterise the networks describing the relationships *within* each time-slice, as well as those *between* time-slices. Analysing the connections between variables at one time-point, while assuming that relationships remain the same over time, allows us to characterise the networks within a time-slice more confidently, by pooling together data from different time-points. On the contrary, analysing the links between variables at adjacent time-points also enables us to discover the relationships between slices. Situations where we have a variable *A* at time *T* affecting a variable *B* at time *T* + 1, and *B* at time *T* affecting *A* at time *T* + 1 are suggestive of a feedback loop.

From a conceptual perspective, we learn a model by jumping through different networks and evaluating a score indicating how well each structure explains the data. However, rather than choosing a single model we follow a Bayesian approach where we account for all models in a measure that is proportional to their score. From each model, we then derive putative causal effects resulting in a distribution which accounts for the uncertainty in the network structure (an approach we first developed in Moffa et al., 2017). Finally, we also take into account the uncertainty in the parameter estimation of each DAG to derive the full posterior distribution of intervention effects (akin to Kuipers et al., 2019).

To explore the space of DAG structures of DBNs in a Bayesian fashion in the current analysis, we relied on our partition Markov chain Monte Carlo method (Kuipers & Moffa, 2017) for sampling DAG structures, and used the implementation for DBNs in the R package BiDAG (https://CRAN.R-project.org/package=BiDAG). To predict the range of causal effects we also followed the Bayesian strategy implemented by Moffa et al. (2017) and Kuipers et al. (2019) for binary variables, using the R package Bestie (https://CRAN.R-project.org/package=Bestie). The main underlying assumptions are that a causal link exists between the variables, that a causal DAG can describe their causal relationship and that all common causes are represented on the DAG (i.e. there are no unmeasured confounders). The method features two special characteristics. First, it informs both the *strength* and the *direction* of causal effects. Second, it captures the uncertainty in the inference that derives from the fact that several different graphical arrangements of variables may each be capable of explaining the data reasonably well, by sampling from the range of possible DAGs in proportion to their posterior distribution. In total, we drew a sample of 10 000 DAGs, in such a way that better fitting graphs appear more often.

Each of the DAGs sampled from the posterior distribution represents a model of the data. The individual DAGs allow us to calculate the effect of one variable on another. Specifically, we can derive an estimate of the probability of each variable taking the value 0 or 1 (depending on the state of its parents in the network), along with the posterior distribution of that probability. The value of one variable is set to 0, and the probability that each remaining variable is either 0 or 1 can then be calculated. The selected variable is then set to 1 and the process is repeated. In Pearl's (2009) terms, the difference between the two sets of values constitutes the causal effect of the selected variable on the others. This procedure is followed in turn for each variable in the DAG. By collating the estimated effects over all the DAGs in the sample we can then obtain the overall effect of changing each variable on all the others. This enables us to identify the most plausible causal mechanisms.

## Results

**Fig. 1.**
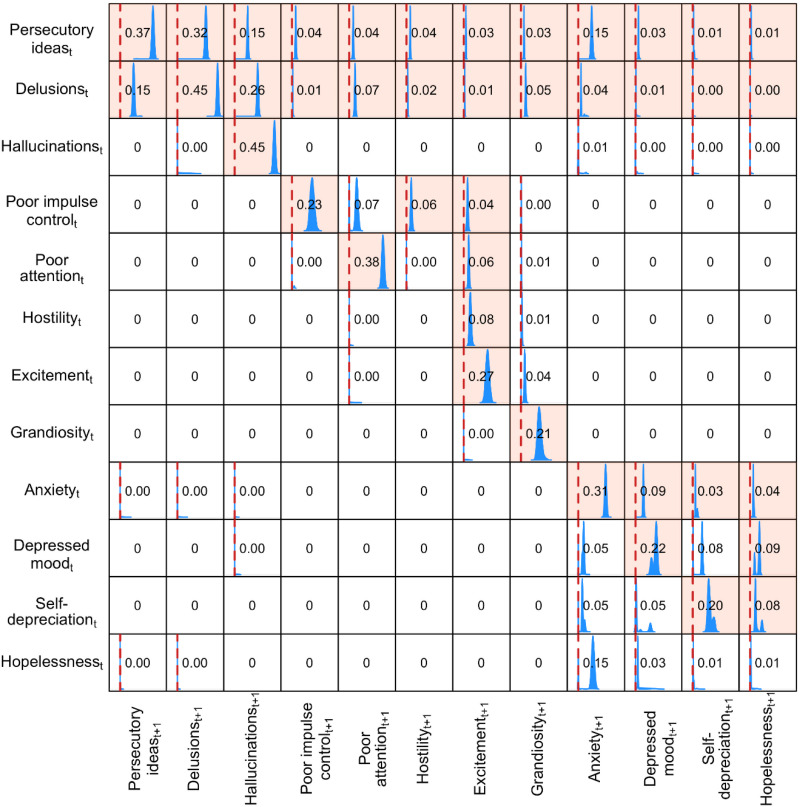
Distributions of causal effects between variables in adjacent time-slices. Each cell displays the distribution of causal effects of row labels (variables from the prior time point) on column labels (variables from the subsequent time point). For readability we truncated the distribution to the range −0.1 to +0.5. The red line in each box represents zero causal and the box is coloured if the 95% credible interval does not straddle the zero causal effect line, to highlight a statistically significant effect. The numerical value in each box indicates the posterior mean of the effect distribution, which gives an estimate for *average causal effect* (zero in the case of no effect). For example enforcing persecutory ideas would seem to increase the probability of delusion at the following time point by 0.32 on average, whereas hallucinations do not show consistent evidence of downstream effects on other variables at the next time point.

**Fig. 2.**
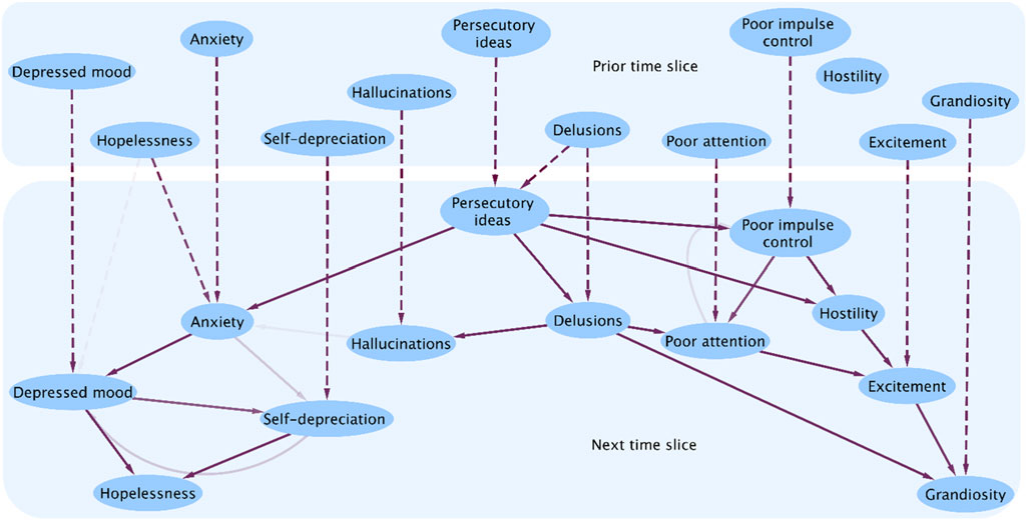
DAG of relationships between variables at initial and subsequent time-slices. Dashed arrows represent the direct relationships between variables from one time-slice to the next. Continuous arrows indicate putative causal links within a time-slice. The density of the arrows represents the strength of the connections. Single-headed arrows correspond to causal links whose direction could be identified under the no unmeasured confounders assumption. Double-headed arrows on the other hand imply that the data are not sufficient to identify a causal direction. The presence of doubly directed links leads to the presence of bimodal peaks in the distribution of causal effect, as we can see in Fig. 1, for example for the effect of depressed mood at time *T* on hopelessness at time *T* + 1, where we can easily recognise two peaks. The larger peak corresponds to structures including the causal path going through the edge from depressed mood to self-depreciation, increasing the total effect. The smaller peak corresponds to the relatively fewer structures where we only have the reversed edge between self-depreciation and depressed mood, leading to a single causal path (depression at time *T* → depression at time *T* + 1 → hopelessness at time *T* + 1) and a smaller total effect.

Figure 1 shows the distribution of causal effects of row labels on column labels: variables from the prior time-slice occupy the rows, whereas those from the subsequent time-slice occupy the columns. Figure 2 shows the DAG corresponding to these findings. The DAG analysis clearly demonstrates that the structure of relationships between symptoms is complex beyond the scope of multivariable logistic regression: the DAGs enable the direction of several effects to be displayed simultaneously.

It is apparent from these figures that most symptoms have *momentum*, that is, they self-predict over time. Indeed, this was the most consistent relationship, and the only symptoms not following this pattern were hopelessness and hostility, implying that these phenomena are short-lived. However, the most striking finding in Fig. 2 is the demonstration of the strong central role of delusions and persecutory ideas, whether by direct or indirect routes. Their persistence in subsequent time-slices enables them to influence the presence of hallucinations at follow-ups. Initial hopelessness influences the presence of later hopelessness, but does so indirectly by increasing anxiety, which in turn has depressive consequences. These then go on to elicit a sense of hopelessness.

Only delusions and hopelessness directly influence symptoms other than themselves over these 6-month intervals: delusions operating on persecutory ideas, and hopelessness on anxiety. Grandiosity is also of interest, as it is the end point of several causal chains: leading from *delusions* via poor attention and excitement, or from *persecutory ideas*, either directly via hostility and excitement, or via poor impulse control, hostility and excitement. In this cohort, hallucinations are either driven by previous hallucinations, or by delusions. There is no evidence that either of these classically psychotic symptoms is driven by affect in this cohort of people with longstanding schizophrenia.

## Discussion

Participants in EuroSC were recruited from patients engaged in established programmes of treatment for schizophrenia, involving monitoring, support and the prescription of medication. They were only followed up in the EuroSC programme if they remained in treatment. Recently hospitalised subjects were excluded, so the sample was not acutely unwell. However, their illness was longstanding, averaging 16 years in duration. The diagnosis of schizophrenia and the assessment of positive and negative symptoms were based on systematic semi-structured interviews. Although (as noted above) symptoms of all types declined in frequency over the study period, the decline was modest, consistent with the fact that participants were not chosen on the basis of a current acute episode. This large and representative cohort, assessed over a period of 2 years at 6-month intervals, provides our study with particular strengths. Retention of participants was good, and distortion of the sample from attrition relatively limited.

Because study participants had generally been treated consistently with antipsychotic medication (Table 1) it is very difficult to discern the effects of medication on their symptoms, in particular because the positivity condition in causal inference requires that people not receiving treatment are included. This was confirmed by the fact that when we included drugs in the structure learning procedure, we found them disconnected from the network of symptoms. This implies further that decisions about treatment tend to be driven by factors unrelated to symptoms, such as side effects. For this reason, we did not pursue a version of DAG incorporating medications

Our most consistent results relate to the connection of symptomatic measures with themselves over a considerable period. It appears that, once formed, symptoms seem to have a degree of momentum that is appreciably independent of the persistence of other symptoms. This finding has not been reported before, and appears to be a significant mechanism underpinning the overall stability of the syndrome of schizophrenia in this sample. It should be emphasised that this occurred in the context of support from community mental health teams and the consistent provision of antipsychotic medication; however targeted psychological treatments were not generally available at the time of the survey.

The longitudinal design provides the basis for our DAG analysis, a novel application in this context. The concept of cause demands a temporal element, in that causes must be antecedent to effects. However, the appropriate period of effect is not intrinsically identifiable. The capacity to demonstrate cause in a longitudinal convenience sample is hostage to the assessment interval: if this is too long, effects may be masked, if too short, there may not have been time for the effect to occur. Experience sampling studies indicate that it is highly probable that causal interactions between psychological processes occur over periods of hours or days (Delespaul, deVries, & van Os, 2002; Myin-Germeys, Delespaul, & van Os, 2005). Such short-term effects may also be cumulative, and remain apparent over longer periods (Fowler et al., 2012), although they may be disguised in a combination of autocorrelation and cross-correlation.

Our five-point analysis of psychotic symptoms is based on the analysis of repeated presentations of the PANSS. This instrument does not provide a specific category of persecutory delusions: delusions are identified phenomenologically, whereas persecutory themes are assessed in a separate item. Some delusions may therefore have non-persecutory content. Equally, persecutory ideas are not necessarily delusional. Nevertheless, it is apparent from Fig. 2 that there is indeed a reassuringly close overall relationship between delusional processing and persecutory ideation. Time 1 delusions predict delusions and persecutory ideation at time 2, time 1 persecutory thinking predicts itself at time 2, and its persistence in turn predicts delusions at time 2. This relationship turns out to be important for the maintenance of two distinct groups of symptoms over the 6-month gap between time-slices. Thus, directly and indirectly, delusions and persecutory ideation result in anxiety, depressed mood, self-depreciation and hopelessness, while a second, derivative, group comprises poor attention, hostility, excitement and poor impulse control, through which grandiosity may be a further consequence. In these data hallucinations appear to be unrelated to other symptoms apart from delusions, and they seemed to be the consequence of delusions rather than the reverse.

Our initial hypothesis was based on the idea that affective disturbance would be causally related to psychotic symptoms, corresponding to their apparent role in acute psychotic episodes and in the development of psychosis in relation to early trauma (Gracie et al., 2007; Hardy et al., 2016; Kelleher et al., 2013; Marwaha & Bebbington, 2015; Moffa et al., 2017). However, counter to our predictions, we did not find this in the current sample. Psychotic symptoms predicted each other, whereas affect appeared to be downstream, in line with traditional views of the relationship between psychotic and affective symptoms. This suggests that, in this stable sample with longstanding disorder, the relationships between symptoms change, such that psychotic symptoms drive affective consequences, particularly depression and anxiety. We did not have in our sample people who were at the onset of their disorder, when affect may be a much more crucial driver of psychosis symptoms (Bebbington et al., 2013; Bird et al., 2019; Freeman, 2016; Freeman et al., 2019; Hardy et al., 2016; Marwaha et al., 2014). Although in the early phases of disorder there may be a precursor affective cascade of anxiety and depression, our results indicate that this is not so in the case of longstanding disorders.

These results suggest that delusional processes are key to the maintenance of the range of psychotic disturbance seen in this cohort of people with longstanding schizophrenia. It is consistent with the specific targeting of delusions in treatment. There is now a growing body of evidence that positive symptoms unresponsive to vigorous pharmacological treatment may be ameliorated by psychological treatments aimed at disrupting the processes involved in the maintenance of delusions (Garety et al., 2008; Kuipers, Yesufu-Udechuku, Taylor, & Kendall, 2014). A recent study has shown that a *blended* (i.e. with combined therapist and digital input), targeted therapy for paranoid ideas was able to improve established delusions (Garety et al., 2021).

### Limitations

As noted, diagnosis was based on information obtained from instruments that differed marginally in their approach to the evaluation of symptoms: SCAN in the UK and Germany and SCID in France. The measures of affect, using the CDSS, were not elaborate. The fact that this was a long-term sample, recruited at a time of stability, may have minimised interactions between symptoms; the assumption of stationarity over time may also have constrained our interpretation of the results. Although the type of medication was identified in the survey, we subsequently excluded it from the analyses presented here because it did not interact with any of the symptoms, either affective or psychotic; this might suggest that by this stage medication effects on mental state had plateaued. Finally our findings inevitably remain hostage to unmeasured confounders.

## Conclusion

In this longitudinal investigation of people with persistent psychotic and affective symptoms over a 2-year period, delusions seemed to be the main drivers of affect (depression, anxiety and grandiosity). Hallucinations appeared to be the consequence of delusions, with no separate affective drivers. Medication did not appear to modify symptom patterns. These results suggest, as has been argued elsewhere, that in longstanding psychosis, further improvement might perhaps be achieved through psychological interventions, such as those recommended in the UK by NICE (2014).

## Supplementary material

For supplementary material accompanying this paper visit https://doi.org/10.1017/S0033291721002920. click here to view supplementary material
