# Supplementary material for: Longitudinal symptomatic interactions in long-standing schizophrenia: a novel five-point analysis based on directed acyclic graphs
Source: Psychol Med. 2021 Aug 5;53(4):1371–8. doi: 10.1017/S0033291721002920 (PMC10009394; doi:10.1017/S0033291721002920)
Supplement: Supplementary file 1 [file S0033291721002920sup001.pdf]

Supplementary Material for:

Longitudinal symptomatic interactions in long-standing schizophrenia: a novel five-point analysis based on Directed Acyclic Graphs

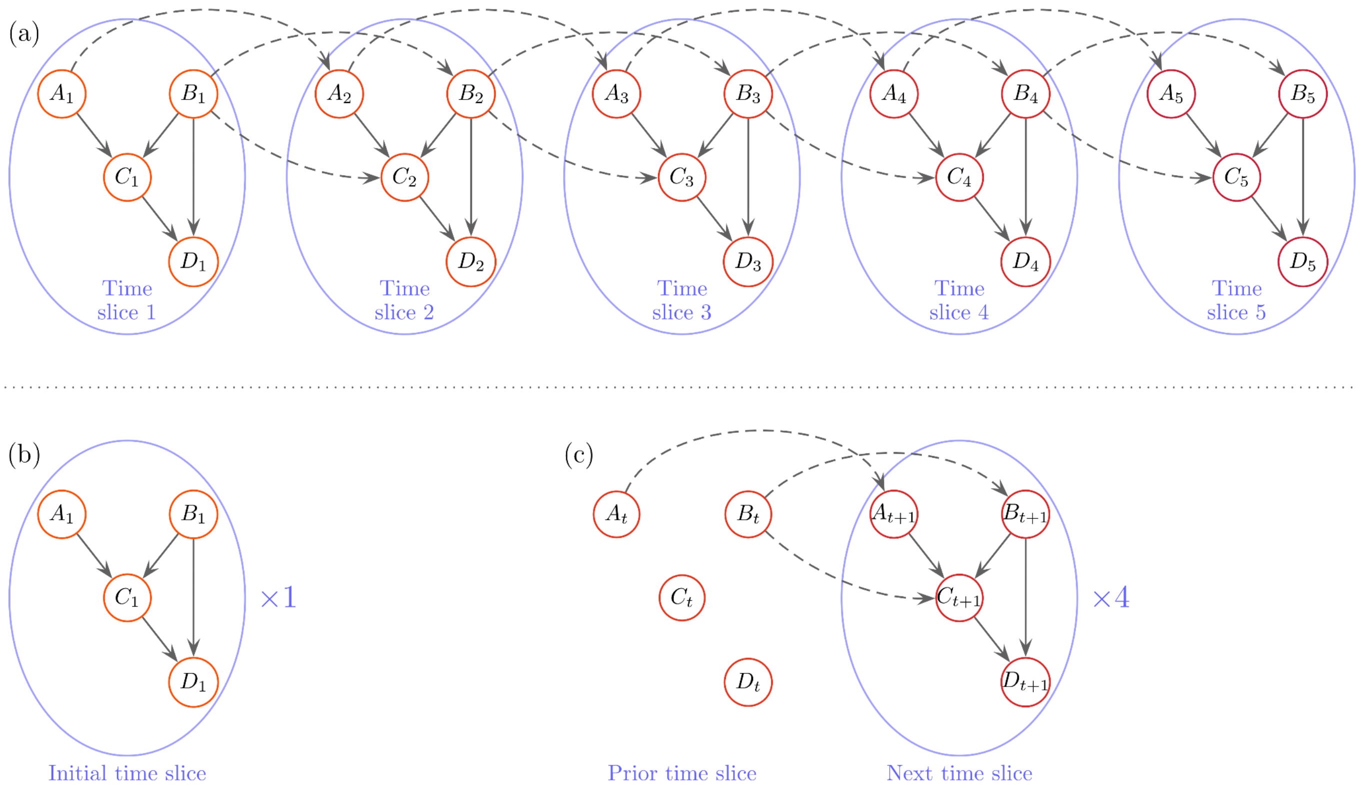

Figure S1: Representation of dynamic Bayesian networks (DBNs). In the example DBN we have 4 variables (A, B, C and D) measured at 5 time points (a). The nodes of the network are the variables at each time point, while the edges indicate the dependencies between variables. The network structure is assumed to be the same within each time slice, and with the same connectors between time slices. The full network (a) can then be more compactly represented as an initial time slice, where there are no connections arriving from before (b), and four copies of the same network between variables within each time slice with edges arriving from the previous time slice (c). From the compact form in (c), one can expand by making four overlapping copies and including the initial time slice from (b) to recover the version in (a). Leveraging the compact form in (c) allows the DBN to be inferred from data at much lower computational cost.

In the learning procedure each possible structure of a DBNs on the given variables (in this example: A, B, C and D at all time points) receives a score indicating how well a model with that particular structure explains the data. By following a sampling procedure we can build a collection of networks where each structure appears to an extent which is proportional to its score. Applying Pearl's *do calculus* to each network in the collection we can derive putative causal effects of each variable on any other. Putting together the effects derived from all the networks in the collection we can get a distribution of plausible causal effects. For the validity of the derived effect we require that there are no important unmeasured confounders.

Initial time slice	Any adjacent time slices	Time slices 1 and 2	Time slices 2 and 3	Time slices 3 and 4	Time slices 4 and 5
1181	1050	989	883	788	726

Table S1: Number of unique individuals with complete data included at the different time points. The total number of observations used for network learning is 1181 for the first time slice and 3386 for adjacent time slices (the sum of the last four columns).
